# Supplementary material for: Serum Glucose-6-Phosphate Dehydrogenase Activity as a Biomarker for Gastric Cancer Stage Prediction
Source: Cancers (Basel). 2025 Nov 27;17(23):3798. doi: 10.3390/cancers17233798 (PMC12691029; doi:10.3390/cancers17233798)
Supplement: Supplementary file 1 [file cancers-17-03798-s001.zip › cancers-3998203-supplementary.pdf]

cancers MDPI

Table S1. Comparison of serum G6PD activity by cancer stage (I-II vs. III-IV) after gender stratification

	Stage I-II	Stage III-IV	p-value
Male	9.0 (8.3-10.8)	13.1 (10.3-14.0)	0.007
Female	10.8 (9.5-12.2)	11.9 (9.8-13.4)	0.367

Data are shown as median (interquartile range, IQR) for G6PD activity. P value was obtained using the Mann-Whitney U test.

Figure S1. ROC curve analysis of G6PD activity stratified by gender (Male: Left, Female: Right)

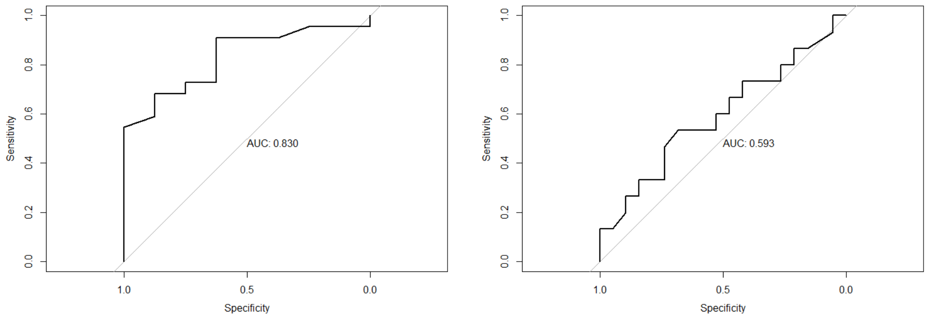

 https://doi.org/10.3390/cancers17233798
